# The Illusory Health Beliefs Scale: validation using exploratory structural equation modeling and multidimensional Rasch analysis

**DOI:** 10.3389/fpsyg.2025.1491759

**Published:** 2025-05-07

**Authors:** Andrew Denovan, Neil Dagnall, Kenneth Graham Drinkwater

**Affiliations:** ^1^School of Psychology, Liverpool John Moores University, Liverpool, United Kingdom; ^2^School of Psychology, Manchester Metropolitan University, Manchester, United Kingdom

**Keywords:** exploratory structural equation modeling, illusory health beliefs, Illusory Health Beliefs Scale, multidimensional Rasch analysis, questionnaire validation

## Abstract

The Illusory Health Beliefs Scale (IHBS) is a multidimensional instrument that evaluates endorsement of scientifically unsubstantiated, illusory health-oriented notions. These beliefs are important because they potentially influence attitudes/actions to the detriment of personal wellbeing/health. Preceding research examining IHBS item performance at the unidimensional subscale level identified five dimensions (Religious/Spiritual, Superstition, Precognitive, Health Myths, Skepticism), and an independent Health Pseudoscience subscale. The present paper extended latent structure analysis by employing exploratory structural equation modeling (ESEM) and multidimensional Rasch analysis. Concurrently, statistical appraisal tested convergent validity via relationships with related health-based constructs (i.e., health locus of control, HLC and beliefs about complementary and alternative medicine, CAM). A sample of 2,138 completed the IHBS (1,016 males, 1,113 females, seven non-binary, two preferred not to disclose). Following minor scale modification, ESEM reported good data-fit for a six-factor model. With the exception of Skepticism, which was negatively associated, IHBS subfactors correlated positively with HLC and CAM. These outcomes supported the supposition that the IHBS measures perceived and illusory health control. Rasch analysis designated sufficient multidimensionality and satisfactory subscale functioning. Strong associations indicated that IHBS dimensions assessed related but discrete aspects of illusory health beliefs. High associations among paranormal-based dimensions (Religious/Spiritual, Superstition, and Precognitive) suggested the need for greater content separation. Moreover, the poor reliability of Skepticism designated the need to develop a more efficacious assessment of this dimension.

## Introduction

Epistemically unwarranted beliefs (EUBs) (e.g., paranormal, pseudoscientific, and conspiracy) are endorsed without support from reliable or credible data (Torres et al., 2022). Despite lacking scientific verification, EUBs are widely held in contemporary societies. Illustratively, belief in the paranormal and conspiracies is common within modern Western cultures (Dagnall et al., 2016, 2022; Drinkwater et al., 2012). Subsumed within EUBs are illusory health beliefs (IHBs). These denote the substantiation of false/ill-informed views, concepts, and perceptions about wellbeing/treatment. Though IHBs are not necessarily harmful to health, they can prove detrimental to personal and social welfare when they influence attitudes and motivate actions that conflict with prevailing medical advice (Capone, 2016). Examples include reduced help-seeking behavior (e.g., lower likelihood to engage with recommended inoculation/treatment and seek advice or support from formal healthcare in response to a problem). Indeed, IHBs can initiate unsafe behaviors such as engagement with ineffective medical treatments and/or therapies (MacFarlane et al., 2018).

Acknowledging these factors, Denovan et al. (2024a, 2024b) developed the Illusory Health Beliefs Scale (IHBS). The inspiration for this measure was the Paranormal Health Beliefs Scale (PHBS) created by Petrillo and Donizzetti (2012) and Donizzetti and Petrillo (2017). The PHBS originated from the observation that supernatural credence embodies specious notions that can potentially negatively affect health-related judgments.

Accordingly, paranormal health beliefs are best delineated as ideations that casually attribute wellbeing/ill-health to unknown powers, forces, energies, and entities, which surpass the limits of what is considered physically possible by current scientific knowledge. From this perspective, paranormal health beliefs within general populations represent self-serving delusions that influence judgments about personal and general welfare (Donizzetti and Petrillo, 2017; Drinkwater et al., 2021; Irwin et al., 2012a,b).

To create the PHBS, Petrillo and Donizzetti (2012) generated content-relevant items, which they then administered to a sample of adolescents. Assessment of responses, using exploratory and confirmatory factor analysis, identified five factors: Religious (protection/recovery allied to higher spiritual forces), Superstitious (practices instigated to ward off threats), Extraordinary Events (mysterious phenomena such as powers/forces), Parapsychological (psychic energies), and Pseudo-Scientific (risks arising from socially deviant/marginalized groups). Psychometric evaluation of the PHBS revealed acceptable performance (i.e., internal reliability and discriminant validity). Hence, Petrillo and Donizzetti (2012) concluded that the PHBS was an effective tool for appraising illusory health beliefs. In a subsequent validation study, Donizzetti and Petrillo (2017) replicated factorial structure and confirmed convergent and discriminant validity.

Despite validation and the potential impact of supernatural beliefs on wellbeing, scholars have made only limited use of the PHBS (e.g., Rosa, 2018). Noting this, Denovan et al. (2024a) examined the utility of the instrument using cognitive interviewing. Participants verbalized their thoughts as they interacted with items. Responses provided insights into perceptions of scale content. Analysis identified problems with cultural specificity, item phrasing, and limited coverage of construct breadth.

These issues stemmed largely from the fact that the PHBS drew heavily on the author’s native, Italian culture. Consequently, subject matter strongly referenced iconic social (i.e., evil eye) and religious (i.e., holy figures) representations. Concomitantly, translation from Italian to English undermined subtlety of expression and resulted in a loss of precision/clarity. Illustratively, items failed to explicitly connect unspecified forces/energies to the paranormal. Finally, interviewees reported that the scale lacked coherence because items indexed specific, eclectic, phenomena. To address these issues, Denovan et al. (2024a) modified PHBS items. They then assessed the effectiveness of their adaptions using a second round of cognitive interviews. Analysis revealed that though changes improved clarity, concerns remained about the extent to which featured phenomena was paranormal.

Consequently, Denovan et al. (2024a) widened the scale’s remit to include illusory (i.e., false) and pseudoscientific beliefs (e.g., advocacy of osteopathy) alongside supernatural credence. This reconceptualization addressed concerns about the classification of paranormal phenomena and facilitated the measurement of propositions that present as scientific but are contrary to prevailing scientific principles. Explicitly, “use non-scientific evidentiary processes including authoritative assertion, anecdotes, or unelaborated ‘natural’ causes” (Losh and Nzekwe, 2011, p. 579). In recognition of these amendments, Denovan et al. (2024a) renamed the modified instrument the Illusory Health Beliefs Scale (IHBS).

To psychometrically appraise the IHBS, Denovan et al. (2024b) obtained responses from a large, UK-based representative sample. Exploratory factor analysis (EFA) identified six dimensions: Religious/Spiritual, Superstition, Precognitive, Health Myths, Skepticism, and Health Pseudoscience. The IHBS demonstrated satisfactory internal reliability and convergent validity. Specifically, positive associations with constructs central to the maintenance of illusory health beliefs (i.e., paranormal belief, magical thinking, endorsement of scientifically unverified notions). Additionally, Rasch analysis found good item/person fit and item/person reliability, unidimensionality, and subgroup item equivalence (gender and religious affiliation). These outcomes supported the shift from paranormal to illusory beliefs.

Development of the IHBS is important because the instrument can advise public health practitioners about the nature and incidence of wellbeing misconceptions. This information is crucial since spurious health-related cognitions negatively impact on general health attitudes and behaviors (i.e., undermine engagement with and the course of recommended treatments). Hence, the IHBS can indicate domains where interventions are necessary and designate the type of information required to target misinformation. In this context, the IHBS has the potential to improve health education, encourage evidence-based behaviors, identify vulnerable individuals/populations, and inform the design of effective interventions.

### Present study

This study assessed IHBS latent structure using exploratory structural equation modeling (ESEM). Scrutiny of latent composition with an independent sample is a significant progression for test development (Boateng et al., 2018). ESEM was appropriate because it combines the strengths of traditional confirmatory approaches (i.e., confirmatory factor analysis, CFA) by including error and statistical fit indices, with the flexibility of EFA (i.e., permits cross-loadings) (Marsh et al., 2014). Additionally, the researchers evaluated the validity of the IHBS using health-based measures (i.e., health locus of control, God Locus of Health Control, and beliefs in complementary and alternative medicine, CAM). This was necessary since Denovan et al. (2024b) established IHBS external validity using measures that indexed general empirical orientation (i.e., paranormal belief, magical thinking, belief in scientifically unsupported ideas, and methods of self-referential, intuitive causality).

The researchers selected locus of control because it is an important construct affiliated with endorsement of both health (Kesavayuth et al., 2020) and epistemically unwarranted beliefs (Hoffmann et al., 2022). Locus of control originates from Rotter’s (1954) social learning theory, it denotes the extent to which individuals attribute behavioral consequences to internal or external factors (Rotter, 1966). Attributions derive from causal expectations that develop through experience (Poortinga et al., 2008). Wallston et al. (1976, 1978) applied locus of control to health and produced the Multidimensional Health Locus of Control Scale (MHLC), which assesses the degree to which individuals take responsibility for their welfare.

Within the MHLC subscales measure reliance on internal (i.e., individual responsibility), and external (chance and powerful others, including the influence of agents such as health professionals) factors. High internal signifies the belief that health is controllable and is associated with higher likelihood to engage in health-promoting/maintaining activities, whereas high external assigns wellbeing to variables beyond the individual’s influence (fate, luck, etc.) and is attendant with lower probability of engagement in positive wellbeing practices (Poortinga et al., 2008). The God Locus of Health Control Scale (GLHC, Wallston et al., 1999) measures the external attribution that God is responsible for health status.

CAM refers to procedures (diagnosis, treatment and/or prevention) that complement mainstream medicine by satisfying demands not met by orthodox medicine (Ernst, 2000). Complementary approaches work alongside conventional treatments, whereas alternative replace standard therapies. Based on previous research, the researchers anticipated that IHBS scores would correlate positively with MHLC dimension scores and CAM-related beliefs.

The investigators predicted these relationships because the IHBS concurrently captures different aspects of control (i.e., perceived and illusion). Lack of perceived control ascribes causation to external factors, whereas illusion of control (the tendency for a person to act as if they possess control over uncontrollable events) manifests as internal locus of control (Irwin, 2000). Illusion occurs when individuals have a high desire for control. For instance, they want to address ill-health and feel powerless. Thus, it is possible, as is the case with the IHBS, that high internal locus of control scores signifies illusory control arising from lack of perceived ability to affect health. In this context, individuals are more likely to engage with CAM since turning to unorthodox treatments allows them to attempt to influence uncontrollable health issues. Commensurate with this prediction, CAM endorsement is associated with internal (Synovitz et al., 2006) and external locus of control (Ebel et al., 2015).

Finally, this study extended previous unidimensional Rasch analysis, which confirmed subscales measured single constructs (Denovan et al., 2024b). Although Davey and Hirsh (1991) advocate this consecutive approach, it is restrictive since it uses a fraction of data allied to a dimension (Adams et al., 1997). When a test is multidimensional, this can undermine item stability and person parameter estimates. To account for this limitation, the present paper undertook multidimensional Rasch analysis, which by calibrating dimensions captures the complexity of scales assessing multiple constructs. The technique provides precise and accurate model estimation and extends preceding analysis (Zhang et al., 2014). Explicitly, affords critical information about dimensionality, item performance (e.g., fit/appropriateness, difficulty), and sample targeting (see Duncan et al., 2003).

## Method

### Participants

The sample comprised 2,138 participants (*M*age = 52.55, *SD* = 15.24, range = 18 to 89); 1,016 males (*M*age = 54.47, *SD* = 14.90, range = 18 to 87), 1,113 females (*M*age = 50.89, *SD* = 15.33, range = 18 to 89), seven non-binary (*M*age = 41.0, *SD* = 18.32, range = 19 to 73), and two preferred not to disclose (*M*age = 43.50, *SD* = 2.12, range = 42 to 45). Recruitment used Bilendi, a provider of quality data. The researchers instructed Bilendi to gather a UK sample comprising an equal distribution of gender and age, with a minimum age of 18. Use of participation panels is comparable to traditional approaches (Kees et al., 2017).

### Measures

#### Illusory Health Beliefs Scale (IHBS)

The IHBS (Denovan et al., 2024a, 2024b) assesses advocacy of scientifically unsubstantiated health-oriented beliefs (e.g., ‘Horoscopes accurately predict health’). The instrument contains 41-items measured alongside a 10-item pseudoscientific health beliefs scale [e.g., ‘Physical illnesses can be cured through the manipulation and channeling of forces and energies (Reiki)’]. The IHBS uses a five-point Likert format, from 1 = Strongly disagree to 5 = Strongly agree. For reliability see the Results section. Preceding work, using exploratory factor analysis, identified the existence of five subfactors (Precognitive, Superstition, Religious/Spiritual, Health Myths, and Skepticism) in addition to the Health Pseudoscience subfactor. The work of Petrillo and Donizzetti (2012), Fasce and Picó (2019), and Torres et al. (2020) informed IHBS development.

#### Multidimensional Health Locus of Control Scale (MHLC)

The MHLC (Wallston et al., 1976, 1978) measures three dimensions related to health-based locus of control: internal, chance, and powerful others. Items (e.g., ‘No matter what I do, if I am going to get sick, I will get sick’) use a six-point Likert response format, from 1 = Strongly disagree to 6 = Strongly agree. Moshki et al. (2007) reported reliability estimates of 0.68, 0.66, and 0.72, respectively, for subfactors.

This study also used the six-item God Locus of Health Control (GLHC) Scale (Wallston et al., 1999), which evaluates the belief that God is responsible for health status (e.g., ‘Whatever happens to my health is God’s will’). This uses the same response format as the MHLC. Reported internal reliability is high (Boyd and Wilcox, 2020). In this study, internal (0.78), chance (0.72), powerful others (0.78), and GLHC (0.97) exhibited acceptable omega reliability.

#### Holistic Complementary and Alternative Medicines Questionnaire (HCAMQ)

The HCAMQ (Hyland et al., 2003) appraises beliefs about CAM, using 11-items (e.g., ‘Complementary medicine builds up the body’s own defenses, so leading to a permanent cure’). A six-point response format (1 = Strongly agree to 6 = Strongly disagree) accompanies each item. Internal scale reliability is satisfactory (Aktaş and Bakan, 2021). Omega reliability in this study was 0.66.

#### Procedure

Participants received a hyperlink, containing study information alongside sections on demographics, study measures, and the debrief. Only consenting participants progressed to the online survey. Instructions emphasized the importance of reading items carefully and responding truthfully. Qualtrics inbuilt randomizer rotated scale presentation to reduce order effects. The Manchester Metropolitan University Ethics Committee (EthOS ID #52313) provided ethical approval.

#### Analysis plan

Following data screening, Harman’s single factor test via exploratory factor analysis (EFA) assessed common method bias (CMB) (Denovan et al., 2021; Podsakoff et al., 2003). CMB indicates the degree to which variance is attributable to a measurement method rather than underlying constructs. Typically, variance > 50% is a cause for concern when all items are specified to load onto a single factor. Next, exploratory structural equation modeling (ESEM) examined data-fit of the six-dimension IHBS model. ESEM is superior to competing approaches, such as confirmatory factor (CFA) and exploratory factor analysis (EFA), due to greater flexibility and the inclusion of both CFA and EFA strengths. Specifically, ESEM permits item cross-loadings alongside tests of fit indices (Asparouhov et al., 2015). ESEM estimation used weighted least square mean and variance adjusted estimation (WLSMV), implemented in Mplus v8 (Muthén and Muthén, 2018). Consultation of fit indices included Comparative Fit Index (CFI), Tucker-Lewis Index (TLI), Standardized Root-Mean-Square Residual (SRMR), and Root-Mean-Square Error of Approximation (RMSEA) with a 95% Confidence Interval.

Following tests of external validity with health belief measures (i.e., MHLC, GHLC, and HCAMQ) the supported ESEM solution was subjected to Rasch analysis. This included scrutiny of unidimensionality with Winsteps 3.81 (Linacre, 2014), focusing on a Principal Components Analysis (PCA) of residuals. Eigenvalues > 2.0 indicate that a dimension extracted from the residuals is meaningful and undermines the unidimensionality assumption of the Rasch model (Linacre, 2012). Local independence assumption testing applied Yen’s Q3 statistic. Subsequently, multidimensional Rasch analysis of the IHBS occurred via ConQuest version 5.34 (Adams et al., 2023). Analysis implemented the Monte Carlo method, alongside marginal maximum likelihood estimation. The polytomous item format necessitated application of the Partial Credit Model.

Determination of item fit included Infit and Outfit Mean Square (MNSQ), which compute misfit between data and model. Values between 0.5 and 1.5 are optimal (Linacre, 2015), and values between 1.5 and 2.0 do not degrade measurement unless multiple instances exist (Wright and Linacre, 1994). Consideration of the item-person map determined item-to-sample targeting, followed by examination of person reliability indices, and correlations between latent dimensions.

## Results

### Preliminary analysis

Satisfactory univariate normality existed (i.e., skewness and kurtosis values were between −3.0 and +3.0). Multivariate non-normal skewness and kurtosis occurred: *b1p* (skewness) = 190.0, *p* < 0.001; *b2p* (kurtosis) = 235.45, *p* < 0.001. Estimation using WLSMV functions well in conditions of non-normality (Finney and DiStefano, 2006).

### Harman single-factor test

A single factor accounted for 20.98% of variance (Eigenvalue = 17.62), which is sufficiently below the 50% threshold. This inferred that CMB did not meaningfully impact the variables and the study conclusions.

### Factor analysis

Good fit existed for the six-factor ESEM model, *χ^2^*(940) = 4725.02, *p* < 0.001, CFI = 0.98, RMSEA = 0.04 (90% CI of 0.04 to 0.05), SRMR = 0.02. Standardized parameter estimates revealed items loaded significantly on their designated factor. However, some Precognitive items loaded more strongly on Superstition and Religious/Spiritual. Systematic removal/reallocation of these items occurred, with re-estimation of the ESEM after each instance. A solution with more tolerable cross-loadings existed after removing item 33, allocating items 19, 22, and 27 to Superstition, and items 34 and 35 to Religious/Spiritual. Reallocated items possessed thematic similarity with newly assigned dimensions, hence permission of the changes. Explicitly, items 19 (‘Some psychics can accurately predict illness’), 22 (‘People have visions about things that can affect their health’), and 27 (‘Hunches and intuitions about illness are not just coincidences’) contain features of superstition in the sense they refer to perceived supernatural influence, including psychic prediction and foretelling. Items 34 ‘Some people have a special gift to heal other people from illness simply by touching them’, and item 35 ‘Health conditions can be treated with spiritual healing’ encompass aspects of spirituality and faith, consistent with the Religious/Spiritual dimension. This solution exhibited good fit, *χ^2^*(897) = 4387.73, *p* < 0.001, CFI = 0.98, RMSEA = 0.04 (90% CI of 0.04 to 0.04), SRMR = 0.02.

ESEM factor loadings were acceptable, generally exceeding 0.32 (Table 1). Significant cross-loadings occurred. Average target-factor loadings were good (Precognitive = 0.51, Superstition = 0.58, Religious/Spiritual = 0.70, Health Myths = 0.39, Skepticism = 0.66, Health Pseudoscience = 0.64). Significant inter-factor correlations existed between subfactors apart from Health Myths and Skepticism (Precognitive with Superstition = 0.65, Precognitive with Religious/Spiritual = 0.67, Superstition with Religious/Spiritual = 0.68, Precognitive with Health Myths = 0.28, Superstition with Health Myths = 0.36, Religious/Spiritual with Health Myths = 0.26, Precognitive with Skepticism = −0.16, Superstition with Skepticism = −0.19, Religious/Spiritual with Skepticism = −0.08, Health Myths with Skepticism = 0.01, Precognitive with Health Pseudoscience = 0.33, Superstition with Health Pseudoscience = 0.38, Religious/Spiritual with Health Pseudoscience = 0.48, Health Myths with Health Pseudoscience = 0.14, Skepticism with Health Pseudoscience = 0.27).

**Table 1:** Psychometric properties of the Illusory Health Beliefs Scale at the item level.

Item	Factor	ESEM loading	Infit MNSQ	Outfit MNSQ	Difficulty
1. Illness can be overcome by psychic forces	Precognitive	0.70	1.03	0.96	0.30
2. It is better to avoid medical appointments (for example, visiting the doctor or dentist) on certain dates, such as Friday 13th	Superstition	0.52	1.7	1.27	0.59
3. People can influence health through psychic forces	Precognitive	0.78	0.79	0.82	0.35
4. The soul or spirit can influence health	Precognitive	0.44	0.98	1.0	−0.46
5. Horoscopes provide important health-related information	Superstition	0.61	0.75	0.90	0.38
6. Religious faith heals diseases	Relig./Spir.	0.86	0.67	0.80	0.23
7. Superstitions, such as saying ‘touch wood’ or actually touching wood, ward off threats to health	Superstition	0.54	0.89	0.97	0.17
8. Holy water protects against illness and disease	Relig./Spir.	0.58	0.71	0.90	0.57
9. Cases of healing due to strength of religious faith exist	Relig./Spir.	0.85	0.93	0.98	−0.36
10. Curses may cause illness	Relig./Spir.	0.40	1.18	1.19	0.22
11. States of illness can facilitate the separation of the spirit from the body	Precognitive	0.36	0.68	0.81	0.16
12. During acute health conditions, it is possible to feel that one’s own spirit is floating out of one’s own body or to perceive one’s own body from an external position	Precognitive	0.33	1.38	1.30	−0.47
13. Superstitions associated with bad luck, such as breaking a mirror, have no impact on health (R)	Skepticism	0.65	1.06	1.02	0.14
14. Health is in the hands of God	Relig./Spir.	0.83	1.45	1.34	−0.37
15. Wearing an amulet or a lucky charm helps to keep one healthy	Superstition	0.47	0.77	0.90	0.15
16. Psychic forces can provoke changes in health conditions (such as an increase in body temperature or a quickening of the heartbeat)	Precognitive	0.42	0.73	0.79	0.12
17. The powers of the mind cannot cure people of illness (R)	Skepticism	0.72	1.01	1.0	0.26
18. Only science and modern medicine can explain why people contract illness (R)	Skepticism	0.57	0.97	0.97	−0.04
19. Some psychics can accurately predict illness	Superstition	0.53	1.03	1.05	−0.63
20. I believe that ‘eating an apple a day will keep the doctor away’	Health Myth	0.27	1.13	1.09	0.19
21. It is important to ‘feed a cold and starve a fever’	Health Myth	0.29	0.99	1.01	−0.30
22. People have visions about things that can affect their health	Superstition	0.35	1.28	1.34	−1.12
23. A person’s future health has nothing to do with their zodiac sign (R)	Skepticism	0.69	0.88	0.92	−0.37
24. The power of prayer can cure disease	Relig./Spir.	0.99	0.84	0.94	−0.27
25. Guardian angels or other spiritual forces can protect against illness	Relig./Spir.	0.63	0.84	0.90	−0.01
26. Fortune telling (using a crystal ball, reading tea leaves) can predict future health	Superstition	0.80	0.66	0.80	0.16
*27. Hunches and intuitions about illness are not just coincidences*	Superstition	0.36	–	–	–
28. Horoscopes accurately predict your health	Superstition	0.70	0.70	0.80	0.53
29. ‘Cracking your knuckles’ causes arthritis	Health Myth	0.46	0.97	0.97	0.25
30. Sitting too close to the television will harm your eyesight	Health Myth	0.50	1.08	1.07	−0.43
31. If you ‘catch a chill, you will catch a cold’	Health Myth	0.54	0.89	0.89	0.17
32. Religious faith contributes to a person’s general health	Relig./Spir.	0.91	1.49	1.37	−0.33
*33. It is possible to have visions about becoming ill which come true*	Precognitive	–	–	–	–
34. Some people have a special gift to heal other people from illness simply by touching them	Relig./Spir.	0.50	0.95	1.01	0.01
35. Health conditions can be treated with spiritual healing	Relig./Spir.	0.52	0.77	0.91	−0.01
*36. Breaking glass or a mirror does not bode well for health*	Superstition	0.54	–	–	–
*37. Card reading (tarot cards) can tell a lot about a person and their future health*	Superstition	0.82	–	–	–
38. Healing prayer from a spiritual healer can cure disease	Relig./Spir.	0.66	0.59	0.74	0.33
39. Radiation absorbed from using a mobile phone can cause cancer	Health Myth	0.26	0.97	0.97	0.11
40. Some people can predict your future health by looking at the lines on your palm	Superstition	0.71	0.74	0.87	−0.22
Health Pseudoscience subscale					
1. Possessing a positive and optimistic attitude helps to prevent cancer	Health Pseudoscience	0.38	1.20	1.15	0.65
2. Osteopathy encourages the body to heal itself by manipulating specific muscle tissue and bones	Health Pseudoscience	0.69	0.85	0.87	0.07
3. Physical illnesses can be cured through the manipulation and channeling of forces and energies (Reiki)	Health Pseudoscience	0.54	0.86	0.87	0.95
4. Homeopathic remedies, where individuals are given diluted substances to trigger natural healing mechanisms, effectively complement the treatment of diseases	Health Pseudoscience	0.61	0.86	0.87	0.54
5. Pain problems can be successfully treated by inserting needles in specific parts of the body (acupuncture)	Health Pseudoscience	0.75	0.93	0.94	−0.24
6. Nutritional supplements, such as vitamins or minerals, enhance health and prevent diseases	Health Pseudoscience	0.58	1.08	1.08	−0.55
7. Toxic substances can be effectively eliminated from the body through detox therapies and/or diets	Health Pseudoscience	0.58	0.97	0.98	0.12
8. Stomach ulcers can be caused by stress	Health Pseudoscience	0.64	1.20	1.21	−0.85
9. Chiropractic (manipulation of the joints) is a scientifically supported form of physiotherapy	Health Pseudoscience	0.78	1.00	1.01	−0.37
10. Applying pressure to specific parts of the feet (Reflexology) can relieve pain in connected areas of the body, such as organs, muscles, and joints	Health Pseudoscience	0.86	0.84	0.86	−0.30

Item in italics removed following ESEM or Rasch analysis; (R) denotes reverse-keyed item; Relig./Spir., Religious/Spiritual.

This, alongside significant cross-loadings, supported the appropriateness of ESEM. Omega reliability was acceptable to good (Precognitive = 0.90, Superstition = 0.94, Religious/Spiritual = 0.95, Health Myths = 0.78, Skepticism = 0.66, Health Pseudoscience = 0.87). Correlations between IHBS subfactors and external criteria (MHLC, GHLC, and HCAMQ) were significant and predominantly in the expected direction (i.e., positive) (Table 2).

**Table 2:** Correlations between IHBS factors (including multidimensional Rasch associations) and the variables used to establish external validity.

Variables	Precog	Super	Relig	Myths	Scept	Pseudo
MHLC						
Internal	0.24**	0.24**	0.23**	0.31**	0.18**	0.44**
Chance	0.25**	0.31**	0.25**	0.29**	0.18**	0.28**
Powerful others	0.23**	0.30**	0.27**	0.33**	0.12**	0.26**
GHLC	0.52**	0.58**	0.73**	0.42**	−0.14**	0.25**
HCAMQ						
HCAMQ total	0.42**	0.38**	0.39**	0.29**	−0.24*	0.46**
IHBS (multidimensional Rasch)						
Precog						
Super	0.84					
Relig	0.83	0.81				
Myths	0.72	0.79	0.74			
Scept	−0.08	−0.18	−0.07	0.21		
Pseudo	0.68	0.62	0.63	0.74	0.24	

Precog, precognitive; Super, superstition; Relig, religious/spiritual; Myths, health myths; Scept, skepticism; Pseudo, health pseudoscience; GHLC, god health locus of control; *p < 0.05, **p < 0.001.

### Multidimensional Rasch analysis

Initial assessment of IHBS unidimensionality, using PCA residuals, found the first contrast eigenvalue was 8.2, inferring the presence of multiple dimensions. In addition, Yen’s Q3 revealed nine correlations exceeded 0.20 (the absolute value indicative of local dependence) (Yen, 1984). This further supports the presence of multidimensionality, inferring that more than one latent trait influences responses to items. Accordingly, IHBS dimensions were calibrated. Outfit and Infit MNSQ for all items (apart from 36) fell within Linacre’s (2015) optimal fit range of 0.5 and 1.5. A second analysis without this item reported that items 27 and 37 deviated from this criterion. Removal resulted in optimal MNSQ (Table 1).

**Figure 1:**
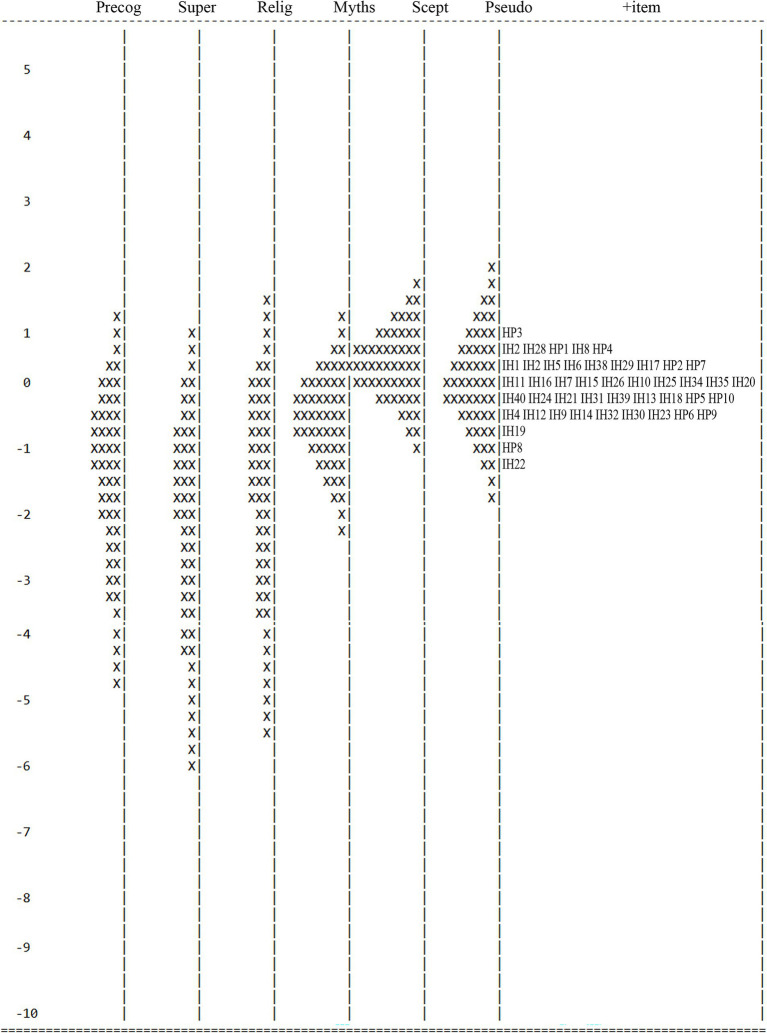
Item-person map for the IHBS. Precog, precognitive; Super, superstition; Relig, religious/spiritual; Myths, health myths; Scept, skepticism; Pseudo, health pseudoscience. The participants are on the left and more able participants are located at the top of the map. Items are located on the right (under ‘+item’) and more difficult items are located at the top of the map. IH, illusory health item; HP, Health Pseudoscience item. Each ‘X’ represents 40.2 cases.

The Rasch model facilitates understanding of item difficulty in relation to a sampled population. The item-person map (Figure 1) illustrated that participants found item 22 (from Superstition) the easiest, and item 3 (from Health Pseudoscience) the most difficult to endorse. This is verified by item difficulty in Table 1, which ranged from −1.12 (item 22) to 0.95 (Health Pseudoscience item 3) logits. Though items clustered closely, the mean of Rasch person measures varied in each IHBS dimension, further supporting multidimensionality (Table 3). The lowest mean existed for Superstition, revealing that this was not strongly endorsed. Indeed, lower means occurred for the paranormal-based subscales (i.e., Precognitive, Superstition, and Religious/Spiritual).

**Table 3:** Mean of person measure.

IHBS subscale	Person mean	Range	
		Minimum	Maximum
Precognitive	−1.48	−0.47	0.30
Superstition	−2.30	−1.12	0.59
Religious/spiritual	−1.74	−0.37	0.57
Health myths	−0.58	−0.43	0.25
Skepticism	0.34	−0.37	0.26
Health Pseudoscience	0.02	−0.85	0.95

Regarding the efficiency of subscales, Person Separation Reliability was 0.73 for Precognitive, 0.78 for Superstition, 0.80 for Religious/Spiritual, 0.72 for Health Myths, and 0.85 for Health Pseudoscience. Lower measurement precision existed for Skepticism, which was below the recommended 0.7 cutoff (i.e., 0.54). Item Separation Reliability scores (i.e., how well the sample could separate items within the IHBS) were 0.99 for Precognitive, Superstition, and Religious/Spiritual, 0.94 for Health Myths, 0.73 for Skepticism, and 0.98 for Health Pseudoscience, reflecting good reliability.

Associations between IHBS dimensions ranged from −0.07 to 0.84 (Table 2). These correlations were corrected for error and were free from measurement ‘noise’ (Briggs and Wilson, 2003). As expected based on the ESEM model, Precognitive, Superstition, and Religious/Spiritual correlated highly. This was due to shared subfactor overlap (i.e., supernatural/paranormal content). The lowest correlations concerned Skepticism, which associated weakly with Precognitive, Superstition, and Religious/Spiritual. This was because Skepticism undermines belief in paranormal phenomena.

## Discussion

Principal objectives of this study included latent structure analysis of the IHBS using ESEM and psychometric performance appraisal via multidimensional Rasch analysis. Additionally, scrutiny of IHBS relationships with related health-based constructs (health locus of control, HLC and beliefs about complementary and alternative medicine, CAM) gauged convergent validity.

High positive correlations between Precognitive, Superstition, and Religious/Spiritual factors illustrated that these subscales assessed core aspects of paranormal belief (Dagnall et al., 2010). Correspondingly, factor content aligned well with principal measurement instruments, the Revised Paranormal belief Scale (RPBS) (Tobacyk, 2004) (i.e., religious belief, spiritualism, psi, precognition, and superstition) and the Australian Sheep Goat Scale (Thalbourne and Delin, 1993) (i.e., extrasensory perception, psychokinesis, and life after death) (see Drinkwater et al., 2017, 2018). However, observed associations were higher than those typically reported and suggested a degree of theoretical conflation. This was not an issue per se since the factors measured primary paranormal beliefs, but it did blur conceptual factor distinctiveness.

The issue arose from shared commonality. In the case of the Precognitive subscale, items indexed a range of supernatural notions (psychic forces, spirit, soul, psychokinesis, etc.), which collectively assess the conviction that psychic and spiritual forces/powers influence health (Denovan et al., 2024b). In this context, the factor label is misleading since precognition traditionally denotes phenomena affiliated with awareness of future events. Hence, this requires revision in subsequent studies.

Overlap between Precognitive and Religious/Spiritual factors was explained by the fact that both factors indexed notions of paranormal/mystical forces (i.e., psychic and soul/spirit). Additionally, Superstition and Religious/Spiritual referenced the desire to resolve uncertainty and lack of control. Noting this, future work should focus on the overarching function of dimensions (i.e., afford meaning, control, and assurance) rather than item content. This high-order, adaption focused approach was previously applied to the RPBS (see Houran et al., 2001).

As predicted, IHBS scores correlated positively with MHLC dimensions, GHLC, and CAM. The only exception was Skepticism, which correlated negatively with GHLC and CAM. These associations supported the supposition that IHBS factors concomitantly assessed perceived and illusion of control. In the case of perceived control, endorsement of illusory health beliefs indicated that individuals considered wellbeing beyond their personal sphere of influence (i.e., governed by external, irresistible factors). This was evidenced by associations between IHBS factors and measures of external control (i.e., MHLC Chance and Powerful Others and GHLC). Regarding illusory control, IHBS dimension relationships with the Internal MHLC and CAM designated desire for control. This was consistent with the prediction that CAM endorsement represents an illusory attempt to influence uncontrollable health issues.

Multidimensional Rasch analysis revealed that three IHBS items misfitted. The remaining items functioned well. Person and Item Separation Reliability designated that subscales, with the exception of Skepticism, performed effectively. Due to possessing few items, Skepticism evidenced less measurement precision. Analysis suggested that the subscale may not be sufficiently sensitive to distinguish between high and low scorers (Boone et al., 2014). Thus, future investigations should revise Skepticism content and generate additional items.

Analysis demonstrated that the IHBS possessed sufficient multidimensionality, evidenced by substantive structure in Rasch residuals (Linacre, 2012) and satisfactory subscale performance. Compared with approaches including classical test theory and unidimensional Rasch, multidimensional Rasch simultaneously assesses relationships between subscales and provides detailed item-level information. Accordingly, improvement of correlation and test reliability accuracy occurs because the estimation includes measurement error (Lee et al., 2010). High correlations among subscales assessing ideations affiliated with paranormal beliefs existed. This was consistent with the ESEM results and inferred that the subscales were empirically related. Moderately strong associations between these paranormal subscales and Myths and Health Pseudoscience demonstrated that IHBS dimensions capture related but discrete aspects of illusory health beliefs. Notably, the misapplication of scientific notions and the propensity to erroneously view unconventional health approaches as scientific. However, the high associations among paranormal subscales revealed the need for greater content separation.

Through Rasch analysis, IHBS items were calibrated from low to high complexity (i.e., easy to difficult). Using the same logit scale facilitated direct comparisons between person ability and item complexity across subscales. The clustering pattern showed the need for more challenging and straightforward items. Moreover, paranormal subscales typically produced lower endorsement. This was due to the polarizing nature of the items, which varied as a function of belief (i.e., ‘believers’ vs. ‘non-believers’). This response pattern was consistent with the observation that supernatural and religious beliefs are widely endorsed within general populations (Williams et al., 2022; World Values Survey, 2023).

### Limitations and implications

The current study indicated that the Skepticism subscale possessed low reliability. This concurs with previous findings (Denovan et al., 2024b). Whether the subscale length or the item contents (or both) accounted for low reliability needs to be explored in subsequent research to obtain a more reliable and valid assessment. This is important given that skepticism is central with regards to scientific/antiscientific belief, and provides a significant counterpoint to EUBs (French, 2015). Indeed, perhaps a useful approach would be to conduct cognitive interviewing similar to Denovan et al. (2024a) but focusing specifically on the skepticism elements of illusory beliefs regarding health. This may help to improve construct validity and data quality (García, 2011).

An additional limitation concerns the sample. Although the study accessed a large sample, with a fairly equal gender split alongside a broad age range, recruitment used non-probability sampling. This approach may not provide an accurate representation of the target population, hence greater demographic controls should be implemented in future. This includes capturing ethnicity and other variables meaningful to illusory health beliefs, including religious affiliation and health status. Validating the IHBS with more varied and detailed samples will facilitate confidence regarding its measurement precision and suitability for the intended population.

Developing the IHBS has significant public health implications. Health misconceptions contribute to poor decision-making, reduced treatment adherence, and increased susceptibility to pseudoscientific practices. By identifying and conceptualizing prevalent health-related fallacies, the IHBS enables researchers and policymakers to design, refine, and implement interventions that address misinformation. Financially, such misconceptions strain healthcare systems by delaying treatment, promoting ineffective remedies, and increasing costs associated with preventable conditions. Socially, they undermine trust in medical professionals, negatively impact health outcomes, and weaken public health initiatives (Lewandowsky et al., 2012). In this context, the IHBS can support the development of strategies that enhance public and patient education while promoting evidence-based health behaviors. Additionally, researchers can use the IHBS to identify at-risk populations, optimize intervention strategies, and steer resource allocation.

Moreover, practitioners can address high score IHBS patients with a range of strategies that focus on improving decision-making, trust and adherence to evidence-based health behaviors/treatments. Specifically, emphasizing a patient-centered approach and communication; producing simplified treatment regimens (e.g., reduce medication burden, optimize dosing, use compliance aids); provision of detailed and understandable information regarding a high scorer’s condition, treatment plan, and medication management; and keeping in regular contact with the patient to develop rapport and trust (e.g., implementing regular monitoring and follow-ups) (Atreja et al., 2005; Carratalá-Munuera et al., 2022; Zolnierek and DiMatteo, 2009).

Suggestions for future development of the IHBS include, firstly, reliability and validity assessment over time. This could comprise test–retest reliability and longitudinal invariance to establish stability and consistency of the IHBS. Secondly, to conduct a multi-country adaptation of the IHBS to examine cross-cultural invariance. A further avenue for development includes scrutiny across religious groups. Although Denovan et al. (2024b) assessed IHBS invariance/differential item functioning with regards to religious affiliation, this focused on broad categories (i.e., religious vs. no religious affiliation). Lastly, an important development objective involves exploring how IHBS scores relate to health-related outcomes (e.g., medical help-seeking behavior) to determine predictive validity.

## Data Availability

The raw data supporting the conclusions of this article will be made available by the authors, without undue reservation.
